# Detailed cost of robotic-assisted surgery in the Australian public health sector: from implementation to a multi-specialty caseload

**DOI:** 10.1186/s12913-021-06105-z

**Published:** 2021-02-01

**Authors:** Kate McBride, Daniel Steffens, Christina Stanislaus, Michael Solomon, Teresa Anderson, Ruban Thanigasalam, Scott Leslie, Paul G. Bannon

**Affiliations:** 1 RPA Institute of Academic Surgery (IAS), Royal Prince Alfred Hospital and University of Sydney, Sydney, New South Wales Australia; 2 Surgical Outcomes Research Centre (SOuRCe), Sydney, New South Wales Australia; 3 Faculty of Medicine and Health, University of Sydney, Sydney, New South Wales Australia; 4 Sydney Local Health District, Sydney, New South Wales Australia; 5 The Baird Institute, Sydney, New South Wales Australia

**Keywords:** Robotic-assisted surgery, Cost analysis, Public sectors, minimally invasive surgery, Healthcare financing

## Abstract

**Background:**

A barrier to the uptake of robotic-assisted surgery (RAS) continues to be the perceived high costs. A lack of detailed costing information has made it difficult for public hospitals in particular to determine whether use of the technology is justified. This study aims to provide a detailed description of the patient episode costs and the contribution of RAS specific costs for multiple specialties in the public sector.

**Methods:**

A retrospective descriptive costing review of all RAS cases undertaken at a large public tertiary referral hospital in Sydney, Australia from August 2016 to December 2018 was completed. This included RAS cases within benign gynaecology, cardiothoracic, colorectal and urology, with the total costs described utilizing various inpatient costing data, and RAS specific implementation, maintenance and consumable costs.

**Results:**

Of 211 RAS patients, substantial variation was found between specialties with the overall median cost per patient being $19,269 (Interquartile range (IQR): $15,445 to $32,199). The RAS specific costs were $8828 (46%) made up of fixed costs including $4691 (24%) implementation and $2290 (12%) maintenance, both of which are volume dependent; and $1848 (10%) RAS consumable costs. This was in the context of 37% robotic theatre utilisation.

**Conclusions:**

There is considerable variation across surgical specialties for the cost of RAS. It is important to highlight the different cost components and drivers associated with a RAS program including its dependence on volume and how it fits within funding systems in the public sector.

**Supplementary Information:**

The online version contains supplementary material available at 10.1186/s12913-021-06105-z.

## Background

Since its introduction in 2000, robotic-assisted surgery (RAS) using the *da Vinci* Surgical System has become increasingly prevalent worldwide [1]. In Australia, it has been predominantly adopted by urology, gynaecology and most recently, general surgery in the private sector [2]. Several studies have reported the main technical advantages of the technology [3, 4] as well as the safety and efficacy of RAS demonstrating comparable outcomes to those of laparoscopic or open surgery, although the longer-term oncological outcomes have been questioned [5–8]. Now utilising the fourth generation of the system, its evolution is arguably still in its early stages and further iterations may be required to significantly advance the technology. While ongoing developments are vital to ensuring its potential is fully realized, one of the main barriers, particularly in the public sector, continues to be the high costs associated with RAS when compared to equivalent laparoscopic or open procedures.

In Australia, the cost of RAS has been compared to non-robotic procedures for colorectal surgery [9], prostatectomy [10] and mitral valve repair [11]. Similarly, several international studies have investigated the cost reporting great variability [2, 9, 12–17]. These differences may be due to omission of the purchase and maintenance costs, utilisation of varying costing approaches and the application of different annualized costing methods. Furthermore, most of the current studies lack detailed descriptions of the cost drivers including staffing, clinical area, diagnostics or specific RAS costs including capital expenditure, ongoing system maintenance and consumables such as instruments and disposable items.

This inconsistency in approach and lack of detail makes it difficult for local hospital administrators, health ministries and governing bodies to determine whether the costs of the technology are reasonable and worth the ongoing investment, and has the potential to impact on future strategic decision-making. With the recent introduction of the *da Vinci* Xi into a tertiary referral hospital in Australia [18], there is an opportunity to examine the cost of RAS across several surgical specialties in the public sector. Therefore, the primary aim of this study is to provide a detailed description of the implementation cost of RAS and the median per patient cost across multiple specialties. The secondary aim is to describe the main cost drivers for RAS cases and to determine the proportion that RAS specific cost contributes to this.

## Methods

### Study design

This study is a retrospective review of the implementation and surgical episode costs of RAS at Royal Prince Alfred (RPA) Hospital, Sydney. Ethics approval was obtained from the RPA Ethics Committee (protocol number X19–0124 & 2019/ETH08725).

### Surgical robotics program

The surgical robotics program at RPA involves a comprehensive governance framework which covers research, training and operational components, with every RAS patient being enrolled in a research study and undertaken using an existing operating theatre list [18]. All cost data was extracted from the comprehensive surgical robotics program database (called BEST), which contains patient information, surgical reports, quality of life and cost data from a range of sources. To consider the impact of case volume across all costing components, utilisation of the robotic theatre for each specialty was determined using the theatre list allocations during the study period. Maximum utilisation and the corresponding cost per case was calculated using the average number of robotic cases that could be completed per full day list multiplied by the number of allocated sessions during the time period.

### Robotic cost data

All RAS cases performed at RPA were reviewed from August 2016 to December 2018 (28 months) including benign gynaecology (robotic-assisted hysterectomy and endometriosis), cardiothoracic (step-wise partial robotic-assisted coronary artery bypass grafting), colorectal (robotic-assisted rectal resection) and urology (robotic-assisted radical prostatectomy).

The fixed costs of the program incorporated the implementation and maintenance costs as follows:

#### Implementation cost

The implementation cost of the program involved the purchase of the *da Vinci Xi* and sterilizing equipment (Reliance® Vision Single Chamber Washer Disinfector) in December 2015, which was funded through a generous philanthropic bequest. A state-of-the-art robotic theatre was also completed in November 2017 to better accommodate the technology. The annualised cost of the *da Vinci Xi* and sterilizing equipment was calculated based on the total system cost divided by 10 years (estimated life span). The same calculation was undertaken for the robotic theatre however an estimated life span of 20 years was used. The cost per case was calculated using the annualised cost converted to months divided by the number of cases performed during the respective period.

#### Maintenance cost

A three-year robotic maintenance contract is in place for the *da Vinci* Xi. The maintenance cost per case was calculated based on the total maintenance cost divided by three to get an annualized figure and then divided by the number of cases during the respective annual period.

The variable cost component of the program included the per episode costs as follows:

#### Episode cost

The median cost of each individual procedure was calculated from the annual inpatient fractions (iFRACs) costing review. This is an initial top-down costing methodology examining the expenses of each cost centre, grouping them into cost pools, which are then allocated down to services. These costs are then refined by a bottom-up approach. This includes staff (medical, nursing and allied health), critical care, emergency, diagnostics (imaging, pathology, pharmacology, prosthetics, specialist procedure suites), operating room, ward and other (hotel, non-clinical and on-costs). A detailed description of the above mentioned surgical cost variables is described in Supplementary Table 1. The cost of all robotic consumables for each specialty was also described and captured within the BEST database for each patient. Up to five instruments may be used during the aforementioned procedures. Individual robotic instruments can be used up to 10 times before disposal and as such the per patient cost was derived from the total instrument cost divided by 10.

The National Weighted Activity Units (NWAU) were determined for each RAS patient with the Local Health District price utilized per NWAU within each appropriate financial year to determine the total funding granted per inpatient episode.

### Analysis

All patient characteristic and cost variables were expressed as median and interquartile range (IQR); with categorical variables presented as percentages. The total iFRACs and NWAU costs were compared using the Wilcoxon signed-rank test for each specialty. All statistical analyses were performed with IBM SPSS Statistics (version 25; IBM Corporation, Armonk, NY). All cost variables were reported in Australian Dollars. For all currency conversions, the exchange rate at the final month of data collection within each study was used to convert from US to Australian dollars.

## Results

### Patient characteristics

**Fig. 1:**
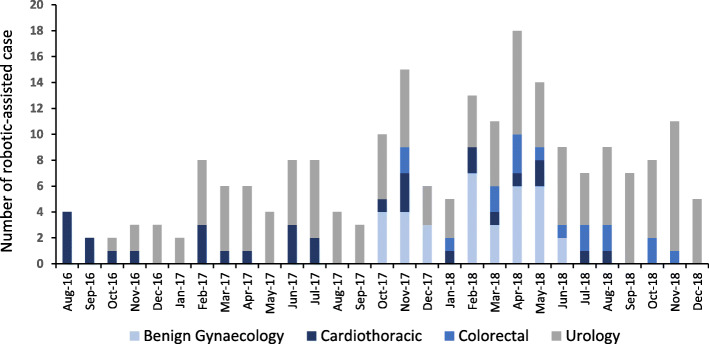
Number of robotic-assisted surgeries by specialty performed during the study period

Between August 2016 and December 2018, 211 patients underwent RAS with an average monthly case volume of 3.4 (Fig. 1). There were 35 (17%) robotic-assisted hysterectomy and endometriosis resections, 31 (15%) partial robotic-assisted coronary artery bypass grafting, 17 (8%) robotic-assisted rectal resections and 128 (61%) robotic-assisted radical prostatectomy. This represents 37% of the maximum volume of RAS cases that could have been performed within the allocated sessions during this period (total 572 cases). The overall median length of hospital stay was 1.9 days, and ranged from 1 day (benign gynaecology) to 6 days (colorectal). The characteristics of the study population are described in Table 1.

**Table 1:** Patient characteristics and detailed costing of robotic-assisted surgery by specialty

	Benign Gynaecology (***N*** = 35)	Cardiothoracic (***N*** = 31)	Colorectal (***N*** = 17)	Urology (***N*** = 128)	Overall (***N*** = 211)
**RAS Patient Characteristics**					
Age, year	43.0 (32.0 to 47.0)	60.0 (54.0 to 68.0)	61.0 (53.0 to 72.0)	64.0 (59.0 to 69.0)	62.0 (53.0 to 68.0)
Sex, Female	35 (100%)	6 (19.4%)	8 (47.1%)	0 (0.0%)	49 (23.2%)
Born in Australia	23 (65.7%)	14 (45.2%)	4 (23.5%)	36 (28.1%)	77 (36.5%)
Private Health Insurance	17 (48.6%)	3 (9.7%)	2 (11.8%)	19 (14.8%)	41 (19.4%)
Employed	21 (67.7%)^a^	10 (55.6%)^b^	10 (58.8%)	48 (42.9%)^c^	89 (50%)^d^
Operating Time (hours)	3.0 (2.7 to 3.5)	7.3 (6.3 to 8.6)	8.3 (7.6 to 8.9)	5.2 (4.9 to 5.8)	5.4 (4.7 to 6.1)
Length of ICU stay (days)	–	2.2 (1.9 to 2.9)^e^	–	–	2.2 (1.9 to 2.9)^e^
Length of hospital stay (days)	1.0 (0.3 to 2.0)	4.0 (2.9 to 6.0)	6.0 (4.8 to 10.3)	1.8 (1.6 to 2.2)	1.9 (1.6 to 3.1)
**Cost variables (AU$)**					
Staff	1184.1 (527.0 to 2062.0)	6420.0 (4366.0 to 7828.0)	5635.0 (3426.0 to 8403.7)	1626.4 (1492.7 to 2144.0)	1728.0 (1477.7 to 3405.0)
Emergency Department	0.0 (0.0 to 0.0)	0.0 (0.0 to 0.0)	0.0 (0.0 to 0.0)	0.0 (0.0 to 0.0)	0.0 (0.0 to 0.0)
Critical Care	0.0 (0.0 to 0.0)	12,403.0 (6828.8 to 16,628.0)	0.0 (0.0 to 0.0)	0.0 (0.0 to 0.0)	0.0 (0.0 to 0.0)
Diagnostic	88.0 (4.0 to 238.0)	4136.4 (2178.8 to 5658.3)	4732.2 (2640.6 to 6851.5)	829.0 (493.4 to 1365.8)	924.1 (358.0 to 2085.6)
Operating Room	6569.6 (3678.1 to 9891.0)	10,655.3 (7528.6 to 13,150.5)	15,387.8 (11,557.6 to 27,165.5)	7891.6 (6852.1 to 9746.6)	8350.0 (6792.0 to 11,352.5)
Ward	6229.0 (3436.5 to 12,417.0)	4489.1 (3825.1 to 6046.5)	4557.1 (3617.4 to 8117.0)	5551.6 (3398.4 to 6153.0)	5469.0 (3643.7 to 6695.4)
Other Costs	1319.0 (507.5 to 2197.0)	5849.8 (2748.3 to 9764.6)	4508.8 (3677.4 to 7018.5)	1915.4 (8,31.0 to 2944.7)	2369.3 (962.7 to 3436.0)
**Total** (iFRACs – AU$)	**15,067.0 (10,101.0 to 24,752.0)**	**44,655.5 (34,432.1 to 61,599.0)**	**32,656.3 (26,528.8 to 57,093.0)**	**17,999.6 (15,201.0 to 21,297.8)**	**19,269.0 (15,445.0 to 32,199.0)**
**Total** (NWAU – AU$)	**6797.1 (5410.5 to 9292.5)**	**45,697.8 (34,537.6 to 50,261.3)**	**21,551.7 (21,237.0 to 22,672.8)**	**15,107.4 (14,868.3 to 15,331.3)**	**15,107.4 (14,729.0 to 21,237.0)**
**Total Variation**	− 8269.90*	**1042.30**	−11,104.60*	− 2892.20*	− 4161.60*

Data presented as median (interquartile range) or N (%); ^a^N = 31; ^b^N = 18; ^c^N = 112; ^d^N = 178; ^e^N = 30 (due to incomplete data). iFRACs = inpatient fractions; NWAU = National Weighted Activity Units; Total variation = Difference between iFRACs and NWAU.*P < 0.005 for the difference between iFRACs and NWAU

### Implementation cost

The implementation cost of RAS included the purchase of the *da Vinci Xi* for $3,900,000 and the sterilizing equipment for $150,000, which from a cash flow perspective was incurred within the financial year 2015/16 prior to the RAS activity commencing, and the theatre refurbishment for $383,186. The total implementation cost was $4,433,186.

During the study period, the implementation cost was $4691 per case. Considering the impact of case volume, the implementation cost would be reduced to $1730 per case if the robotic theatre was completely utilized.

### Maintenance cost

Maintenance of the *da Vinci* Xi was $621,245 for a three-year contract. During the study period, the median maintenance cost was $2290 per case. If the robotic theatre was completely utilized, the maintenance cost would be reduced to $845 per case.

### Episode cost

The overall median cost was $19,269 per case (based on iFRACS data) ranging from $15,067 for benign gynaecology to $44,655 for cardiothoracic (Table 1). The main cost drivers were found within the operating room (44%) and inpatient ward (29%). Operating room costs were the main contributor for urology (44%), benign gynaecology (43%) and colorectal (44%), whereas critical care costs were the highest for cardiothoracic RAS patients (28%).

The total episode cost for each specialty was also calculated using NWAUs, with a mean funding difference of $4162 per case compared to the iFRACS data. There was a significant difference in the total costs for benign gynaecology (*p* < 0.001), colorectal (*p* = 0.003) and urology (*p* < 0.001) (Table 1).

### RAS consumable cost

**Fig. 2:**
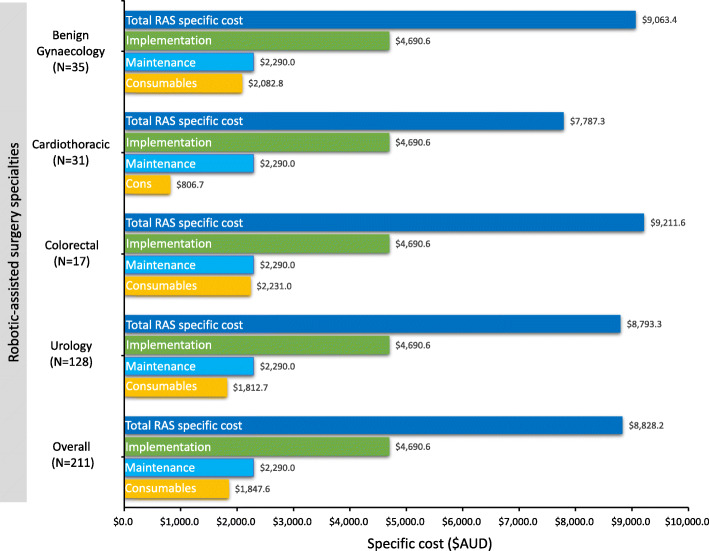
Specific cost of robotic-assisted surgery according to specialty. Total robotic-assisted surgery specific cost: Included implementation, maintenance and consumable costs; Implementation cost: Cost of the da Vinci Xi surgical system, sterilizing equipment and refurbishment of robotic theatre divided by the number of robotic-assisted cases; Maintenance cost: Total maintenance cost divided by the number of robotic-assisted cases; and Consumables cost: Average consumables cost per robotic-assisted case

The RAS specific consumable cost was $1848 per case, ranging from $807 for cardiothoracic to $2231 for colorectal surgery. Overall, RAS consumables contributed approximately 10% of the total cost and 21% of the total RAS specific cost (Fig. 2).

### RAS specific cost

The total costs specifically related to RAS including the implementation, maintenance and RAS consumable costs was $8828 per case ranging from $7787 for cardiothoracic to $9212 for colorectal (Fig. 2**).**

## Discussion

This study provides a detailed description of the median cost per RAS patient for multiple specialties including benign gynaecology, cardiothoracic, colorectal and urology, and outlines the main cost drivers specifically related to RAS such as the implementation, maintenance and consumable costs and considers how these would change depending on case volume.

Substantial variation was found between specialties in line with the different procedure types with the median cost per patient being $19,269 (IQR: $15,445 to $32,199) whereby the overall specific RAS costs contributed almost half of this (46%) A further breakdown of the specific RAS costs found the contribution of the implementation, maintenance and consumable costs to the overall per patient cost were 24, 12 and 10% respectively. It should be highlighted that the fixed implementation and maintenance costs per patient were derived based on the volume of cases undertaken during the study period, which was relatively low. Further investigation is required to determine the reason for this, which could include case selection with an overall robotic theatre utilisation of 37% (ranging from 27% for cardiothoracic to 41% for colorectal). In considering the impact of case volume, it was shown the implementation and maintenance cost can be reduced considerably if the robotic theatre is utilised to its full capacity. This is in keeping with previous research, which has suggested that an increase in the number of cases was associated with cost reduction [13, 19]. Accordingly, implementation costs, which are rarely factored into robotic costing studies, should also be considered from a cash flow perspective regarding when and over what time period the capital costs are incurred by an organization, along with attention being given to how the costs may be further reduced such as not requiring or postponing theatre refurbishments or the negotiation of annual maintenance contracts.

Overall the main cost driver for RAS cases were those incurred within the operating theatre (44%), which is consistent with the findings of other studies [10]. Given it is well known operating theatres are high cost environments regardless of the technology being utilised, an examination of the RAS consumable costs against the overall operating theatre expenditure found it to be contributing to 22% of these costs.

Consideration of the costings described in this study should be made within the context of case selection in terms of the contribution that RAS makes to the overall cost per case. The experience across the four surgical specialties involved in this public hospital was mixed. Within cardiothoracic and colorectal, the cases were complex with patients staying in hospital around 6 days with a median cost of $44,656 and $32,656 per case resulting in the contribution of the additional RAS expenditure of $7787 and $9212 being approximately 17 and 28%, respectively. It is important to note that for some of these cases, usual care would not have been an option due to their complexity. This was in comparison to benign gynaecology and urology where the patients stayed in hospital less than 2 days with the overall cost per case being much lower at $15,067 and $18,000 so the overall contribution of the additional RAS expenditure was much greater at 60 and 49%, respectively. Certainly, the findings from this study clearly outline there are many cost drivers and variables that need to be considered collectively in terms of how a hospital RAS program is to be established and funded. This also extends to broader and longer term policy implications for the uptake of the technology across the public sector in particular. Indeed, within the current Activity Based Funding (ABF) environment, it is also important to highlight the coding system does not recognize RAS resulting in the additional costs of the technology having to be absorbed within the hospital’s general budget. This was evident in the average difference between the iFRAC expenditure data (incorporates all expenditure) and the NWAU costing data (which does not yet recognise robotics) demonstrating an average funding deficit of $4162 per RAS case, which is similar to the specific RAS costs described at $4138 per case (when excluding implementation costs). Although the nationally applied coding system has resulted in this barrier being experienced within RAS programs in other Australian states [9, 10], the funding model has longer term policy implications for the introduction of new surgical technology into the public sector. Arguably the lack of responsiveness and considerable lag time for the coding system to be updated to incorporate new technology or procedures, does not readily support innovation and potentially hinders the public sector having access to the latest technological advances. It should be noted that New South Wales does have a mechanism for supporting models of care involving high cost and low volume patient care; however, surgical robotics is currently not included within this scheme.

The costings described within this study are comparable to other costing studies investigating RAS both nationally and internationally (when converted to Australian dollars). Within benign gynaecology, the per patient cost in this study (AU$15,067) corresponds to the experience in the US for robotic-assisted hysterectomies costed at $AU15,117 [20] and $AU14,906 [21]. Within cardiothoracic, the per patient cost in this study ($AU44,655) for partial robotic-assisted CABG was higher compared to a study in the US where it was found to be $AU18,342 [22]; however, the partial nature of the local RAS cases makes comparison difficult. For RAS within colorectal, the consumable costs were found to be $AU2,728 higher in a Victorian based study [8], which is similar to the $AU2,231 of specific RAS consumables found for rectal resections in this study. Finally within urology, the per patient cost in this study of $18,000 for robotic-assisted prostatectomy was similar to the cost of $AU17,582 found in a Queensland study [10], and $AU13,860 in the US [23].

This study has a number of limitations. Firstly, it is based on the experience of a single centre during a set period of time and there may be some variation in the application of costing methodologies used. Similarly, there are known constraints to using administrative datasets including they have been shown to under-report patient complexity [24]. Although the overall cohort of RAS patients examined is large, the numbers within each specialty are relatively small and may result in the findings not being truly representative. Finally, this study is purely descriptive and does not compare the cost drivers of RAS to conventional laparoscopic or open procedures, or explore the opportunity costs associated with low case numbers. As such the potential cost effectiveness of RAS is not able to be determined from this dataset. However, given the dearth of literature describing the detailed costings of RAS cases in the public sector across multiple specialties it was still felt this study offers a valuable contribution. Future economic studies from this RAS program will be focusing on cost-effectiveness analyses to compare the cost and consequences of RAS versus usual care.

In conclusion, the cost of RAS is substantial contributing between 17 to 60% of the overall cost of the patient’s surgical treatment depending on inclusions, volume and the surgical specialty. It is important for local hospital administrators, health ministries and governing bodies to be aware of the cost components and drivers when establishing a RAS program, and to highlight the importance of new technology being incorporated into standardised funding systems.

## Supplementary Information

**Additional file 1.**

## Data Availability

All data generated or analysed during this study are included in this published article within Table 1 and Figs. 1 and 2, as well as in Supplementary Table 1.

## Abbreviations

ABF: Activity Based Funding
iFRAC: Inpatient fractions
IQR: Interquartile range
NWAU: National Weighted Activity Units
RAS: Robotic assisted surgery
RPA: Royal Prince Alfred
