# Supplementary material for: Detailed cost of robotic-assisted surgery in the Australian public health sector: from implementation to a multi-specialty caseload
Source: BMC Health Serv Res. 2021 Feb 1;21:108. doi: 10.1186/s12913-021-06105-z (PMC7849115; doi:10.1186/s12913-021-06105-z)
Supplement: Supplementary file 1 — Additional file 1. [file 12913_2021_6105_MOESM1_ESM.docx]

| **Supplementary Table 1.** Description of surgical cost variables | |
| --- | --- |
| **Cost variables** | **Definition of each cost variable** |
| **Critical Care** | Cost of all Goods & Services (excluding prosthesis), Salary and Wages and VMO Payments for Critical Care cost centres including ICU, HDU, CTICU, PSICU, NICU, PICU and CCU. |
| **Diagnostic** | Imaging: Cost of all Imaging Goods & Services.  Pathology: Cost of all Pathology Goods & Services.  Pharmacy: Average cost of all Pharmacy Goods & Service costs except for those in Critical Care, SPS, Operating  Prosthetics: Cost of all Prosthesis costs in all cost centres.  Specialist Procedure Suites: Cost of all Good & Services (Excluding Prosthesis), Salary and Wages and VMO Payments for Specialist Procedures Suites (SPS). |
| **Emergency department** | Cost of all Goods & Services, Salary and Wages and VMO Payments for Emergency Department cost centres. |
| **Operating Theatre** | Cost of all Goods & Services (excluding Prosthesis), Salary and Wages and VMO Payments for Operating Theatre cost centres. |
| **Other** | Hotel: Cost of all food and domestic services.  Non-clinical: Amount of non-clinical costs including hotel and administrative costs, non-clinical salaries and wages.  On costs: Amount of on costs. These costs include Superannuation and Workers Compensation premium payments.  Excluded: The average amount of costs that are excluded from the NSW State price including all Depreciation, Annual Leave and LSL actuarial adjustment, Interest.  Patient transport: Average cost of all Patient Transport costs in all cost centres. |
| **Staff** | Allied health: Cost of all Good & Services and Salary and Wages for Allied Health Cost centres.  Medical: Cost of all Medical Salary and Wages and VMO Payments in Clinical Service or Ward cost centres.  Nursing: Cost of all Nursing Salary and Wages in Clinical Service or Ward cost centres. |
| **Ward** | Cost of all Goods & Services for Clinical Service or Ward cost centres. |
